# The Order of Exercise during Concurrent Training for Rehabilitation Does Not Alter Acute Genetic Expression, Mitochondrial Enzyme Activity or Improvements in Muscle Function

**DOI:** 10.1371/journal.pone.0109189

**Published:** 2014-10-07

**Authors:** Lauren G. MacNeil, Elisa Glover, T. Graham Bergstra, Adeel Safdar, Mark A. Tarnopolsky

**Affiliations:** 1 Department of Pediatrics, McMaster University, Hamilton, Ontario, Canada; 2 Department of Health Sciences, McMaster University, Hamilton, Ontario, Canada; 3 Department of Medicine, McMaster University, Hamilton, Ontario, Canada; Universidad Pablo de Olavide, Centro Andaluz de Biología del Desarrollo-CSIC, Spain

## Abstract

Concurrent exercise combines different modes of exercise (e.g., aerobic and resistance) into one training protocol, providing stimuli meant to increase muscle strength, aerobic capacity and mass. As disuse is associated with decrements in strength, aerobic capacity and muscle size concurrent training is an attractive modality for rehabilitation. However, interference between the signaling pathways may result in preferential improvements for one of the exercise modes. We recruited 18 young adults (10 ♂, 8 ♀) to determine if order of exercise mode during concurrent training would differentially affect gene expression, protein content and measures of strength and aerobic capacity after 2 weeks of knee-brace induced disuse. Concurrent exercise sessions were performed 3x/week for 6 weeks at gradually increasing intensities either with endurance exercise preceding (END>RES) or following (RES>END) resistance exercise. Biopsies were collected from the *vastus lateralis* before, 3 h after the first exercise bout and 48 h after the end of training. Concurrent exercise altered the expression of genes involved in mitochondrial biogenesis (PGC-1α, PRC, PPARγ), hypertrophy (PGC-1α4, REDD2, Rheb) and atrophy (MuRF-1, Runx1), increased electron transport chain complex protein content, citrate synthase and mitochondrial cytochrome *c* oxidase enzyme activity, muscle mass, maximum isometric strength and VO_2peak_. However, the order in which exercise was completed (END>RES or RES>END) only affected the protein content of mitochondrial complex II subunit. In conclusion, concurrent exercise training is an effective modality for the rehabilitation of the loss of skeletal muscle mass, maximum strength, and peak aerobic capacity resulting from disuse, regardless of the order in which the modes of exercise are performed.

## Introduction

Muscular adaptation to chronic exercise occurs to maintain cellular homeostasis during future bouts. Typically identified as one of two divergent modes, resistance or aerobic, exercise places stresses upon the myofibre that induce phenotypic changes that differ greatly between the modes. Through sets of low-repetition/high-intensity contractions, resistance exercise promotes hypertrophy and improves anaerobic energy supply, thereby increasing strength/short-term force generation [1], [2]. Alternately, aerobic exercise is characterized by longer periods of high-repetition/low-intensity contractions that promote an increase in oxidative energy capacity, predominately through mitochondrial biogenesis and increased vascularization [3], improving fatigue resistance. However, these changes are not mutually exclusive as both modes modestly affect characteristics associated with the other [4]–[6]. It has been suggested that the adaptive processes may interfere with one another [7], [8], thereby attenuating the optimal adaptation that could be achieved from either mode individually. As such, training strategies for maximum improvement for a specific physical task, such as sport performance, should be designed with one of these endpoints in mind. However, the desired benefits of exercise may include improvements in both strength and aerobic capacity. For example, rehabilitation of changes that occur in skeletal muscle following disuse atrophy [9]–[11] and mitigation of the effects of aging [12], [13] may require exercise programs utilizing both modes of muscle contraction.

Disuse atrophy is the loss of skeletal muscle mass following a reduction or cessation in physical activity that can be the result of factors such as illness, injury, aging, or lifestyle change. Typically, hypodynamia results in a loss of muscle mass [14]–[17], muscle function [14]–[17], and aerobic capacity [17], [18]. Incorporating resistance exercise as a clinical recovery strategy is logical to recover lost muscle mass and strength, whilst aerobic exercise may be beneficial to recover aerobic capacity. Combining both modes of exercise into one program is termed concurrent training. Research into the effects of concurrent training as a method of improving both strength and endurance performance are equivocal. Some studies have found that aerobic exercise attenuates the increases in strength [2], [19], [20], hypertrophy [2], [20] and power [2], [19] that occur with resistance training alone, potentially by the attenuation of signaling through the Akt-mTOR-S6K cascade by interference from AMPK-PGC-1α signaling [21]. However, others have found equal improvements in strength with concurrent training [22]–[24]. The effect of concurrent training on improvements in aerobic capacity when compared to aerobic exercise alone seem to indicate that VO_2_max is unaffected by competing adaptations [25] and may even be beneficial through increased proportions of type IIa fibres [26].

Concurrent training programs are rarely compared for the effect of the order in which the exercise modes are performed. Results from Coffey et al. suggest that the order in which exercise modes are performed may result in divergent adaption during concurrent training [27]. Following up on these findings, we sought to test whether altering the order of exercise would affect the early gene expression patterns and resulting strength and aerobic capacity changes as part of a rehabilitation strategy.

## Materials and Methods

### Subjects and anthropometrics

Eighteen young healthy adults (10 men and 8 women) volunteered as participants in this study. All subjects were screened to ensure they had no pre-existing health conditions and they had not regularly participated in resistance or endurance exercise in the preceding 6 months. Menstrual cycle and oral contraceptive use was not controlled in this study; as such all findings represent general responses to disuse in women. Whole body and body compartment composition and were measured using dual energy x-ray absorptiometry (DXA) scans (Lunar Prodigy Advance, GE Healthcare, Madison, WI). The study subject’s demographics were (mean ± SD): age −20±2 y; height −171±10 cm; weight −70.2±16.9 kg, BMI −24.0±5.2.

### Ethics statement

Each participant was given an information sheet and all of the testing procedures were explained to them before providing written informed consent. The study conformed to the standards outlined in the *Declaration of Helsinki* and was given approval by the Research Ethics Board at McMaster University (REB #09-322).

### Study design

Immediately before beginning this study, all participants had completed 2-weeks of single leg knee joint immobilization with the use of a Rehab post-op knee brace (Össur Americas, Foothill Ranch, CA) that was restricted to 90° knee flexion. Participants returned to the lab daily to have the brace removed, at which time the leg was inspected for pain or edema and were allowed to shower without bearing weight on the treatment leg. The brace was replaced, taped and a unique identifier was used to ensure that knee braces were not removed. Walking crutches were provided to assist with mobility. This protocol has been used previously to induce disuse muscle atrophy and loss of strength [15], [17], [28], [29].

Participants were then randomly assigned to one of two concurrent exercise groups: resistance exercise first followed immediately by endurance exercise (RES>END, N = 9) or endurance exercise first followed immediately by resistance exercise (END>RES, N = 9). Peak aerobic capacity (VO_2peak_), maximum isometric strength and maximum strength (1RM) were tested before immobilization to determine baseline aerobic fitness and strength and to calculate initial rehabilitation exercise values.

VO_2peak_ was measured using a graded incremental cycling test and an Excalibur Sport cycle ergometer (Lode, Groningen, the Netherlands). Following a 5-minute cycling warm up with light resistance, participants began the test at a workload of 50 W and increased by 50 W (men) or 30 W (women) every 2 minutes either until volitional fatigue, defined as the inability to maintain a pedaling frequency above 60 rpm for longer than 5 seconds. Verbal encouragement was provided as participants approached test completion. Heart rate (bpm) and oxygen consumption (L/min) were monitored with a MOXUS modular metabolic system (AEI Technologies, Pittsburg, PA).

Maximum isometric strength of the knee extensors was measured with a System 3 dynamometer (Biodex Medical Systems, Shirley, NY). Participants were seated in the dynamometer, with their knee flexed at 90°, and secured across the thigh, calf, lap and trunk to prevent extraneous movements. All were instructed to maximally contract their knee extensors for 5 seconds. Isometric strength was measured 3 times with a 30 s rest period between each repetition; the highest value (Nm) was recorded for each leg.

1RM was measured as the maximum amount of weight that could be successfully moved through complete range of motion once only for three leg exercises: leg press, knee extension and knee flexion. For each exercise, a brief warm up of 5 repetitions using a submaximal weight and a 1 minute rest was performed before participants attempted 3 repetitions of a weight estimated to be ∼80% of maximum. Weights were increased and single repetitions attempted until movement through the complete range of motion was not possible. The heaviest successful repetition was designated as the 1RM.

### Acute Exercise protocol

Relative exercise intensities and duration were identical between the END>RES and RES>END groups; the only exception between them was the order in which the exercises were performed. Depending on their assigned group participants began with either 22.5 minutes of stationary cycling at 65% VO_2peak_ or 3×10 repetitions of leg press, knee flexion and knee extension at 65% 1RM and immediately transferred to the opposite protocol. The resistance exercise protocol had a 1 minute rest between sets and exercises and was completed in approximately 22 minutes, resulting in a total exercise time of ∼45 minutes.

### Rehabilitative Exercise protocol

**Figure 1:**
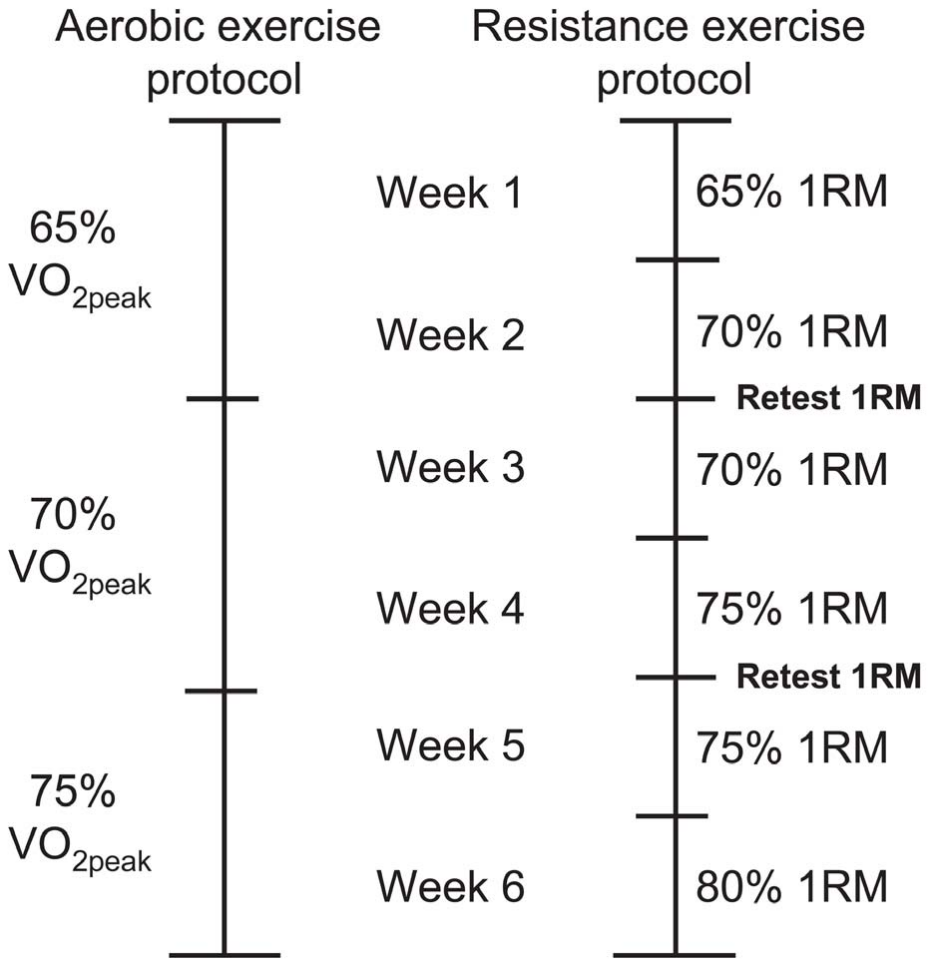
Concurrent exercise program design with weekly intensities for aerobic and resistance exercise protocols.

Participants completed three exercise sessions per week with at least one rest day between sessions. Following the acute exercise protocol, two more identical sessions completed the first training week. Aerobic exercise intensity increased by 5% every two weeks to 70% and 75% of VO_2peak_; exercise duration remained constant at 22.5 minutes. Resistance exercise intensity increased to 70% 1RM for the second week. Maximum strength was retested every two weeks to ensure the progression over the course of the study matched the participant’s rate of strength change. Weeks three and four had intensities of 70% and 75% of the second 1RM test; weeks five and six were 75% and 80% of the third 1RM test (Figure 1).

**Figure 2:**
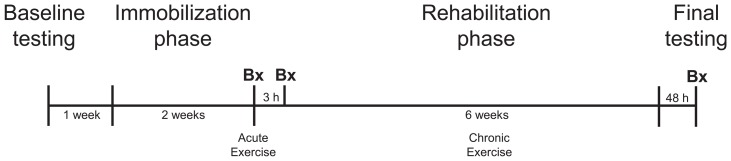
Longitudinal study design. (Bx) - Timing of muscle biopsy.

A muscle biopsy of the *vastus lateralis* was taken under local anesthesia (2% lidocane) using a modified Bergström needle with manual suction from the experimental leg following 2 weeks immobilization (Pre-exercise), 3 hours after the first bout of concurrent exercise (3H) and following 6 weeks of rehabilitative concurrent exercise (Post) (Figure 2). Immediately following excision, the muscle biopsy was dissected free of connective tissue and fat, sectioned into 25–50 mg pieces, placed into 1.5 mL Eppendorff tubes and frozen in liquid nitrogen. Muscle samples were stored at −80°C until processing and analysis. Immediately following each muscle biopsy, blood was collected in BD Vacutainer collection tubes for serum and K_2_EDTA plasma from the antecubital vein and treated following manufacturer’s specifications. Tubes were centrifuged and the supernatant aliquoted and stored at −20°C for future analyses.

### RNA extraction

Total RNA was extracted from skeletal muscle as follows. Muscle tissue (∼25 mg) was homogenized using an electric homogenizer (Pro Scientific) in 1 mL of Trizol Reagent (Life Technologies, Cat. No. 15596, Gaithersburg, MD). The homogenate was then incubated for 10 min at room temperature, followed by phase separation using 200 µL of chloroform and precipitation of the total RNA from the aqueous phase using 380 µL of anhydrous ethanol. The RNA was then isolated using Qiagen RNeasy Mini Kits according to manufacturer’s instructions, eluted with RNase free water, aliquoted, and stored at −80°C. The concentration and purity of the RNA was determined using a Nanodrop UV-vis spectrophotometer (ND 1000; Thermo Scientific, Wilmington, DE) at 260 and 280 nm. The average purity (OD_260_/OD_280_) of the samples was >1.7. Prior to elution, isolated RNA was treated with DNA-free recombinant DNase I (Ambion Inc, Austin, TX) according to the manufacturer’s instructions to remove any potential genomic DNA contamination.

### qPCR analysis

Changes in gene expression relative to baseline values were measured using quantitative real-time polymerase chain reaction (qPCR). Following confirmation of stable expression at all time points, 18S rRNA was used as the housekeeping gene. The efficiencies of all primers were tested and determined to be greater than 98%. The primer sequences can be found in Table 1.

**Table 1:** Primer design.

Gene name	Forward primer	Reverse Primer
18S rRNA	CGGTAATTCCAGCTCCAATAG	CGCTCCCAAGATCCAACTAC
PGC-1α	CATCAAAGAAGCCCAGGTACA	GGACTTGCTGAGTTGTGCATAC
PGC-1β	CCTGTTTATGCCTCCCTCAC	GGTGAAGCTGCGATCCTTAC
PGC-1α4	TCACACCAAACCCACAGAGA	CTGGAAGATATGGCACA
PPARγ	AGAAGCTGTTGGCGGAGAT	CAGCGGGAAGGACTTTATGTA
PRC	CCTACCAAGGTGGAGGTCAA	AGCCTTCATCTGGGGACTTT
REDD1	AGTGCCCTCCAAGACAGAGA	TTAGGTGGCTGCCTCAGTTT
REDD2	GTTCCTGAACCCAACCTCAA	AAGGACCTTTGAGCAACCAA
Rheb	TAGCTCGATGTCCGTGTCA	CAGGCAAGGCTGTTCTCAAT
Atrogin-1	AGCTGGATTGGAAGAAGATGTA	TTTGCAGAGCTGAAGGGTATC
MuRF-1	TCCATGTGCAAGGTGTTTG	GCCACCAGCATGGAGATAC
Nedd4	CAAGGATGAGCACCAGGTATAG	ATGAGGGTGGGCATGATTAC
Runx1	TCAGAGTCAGATGCAGGATACA	ATCCCAGGTATTGGTAGGACTG

qPCR was performed using PerfeCta SYBR Green SuperMix, ROX (Quanta BioSciences, Gaithersburg, MD). The primers to each target gene were designed using cDNA sequences in GenBank (http://www.ncbi.nlm.nih.gov/sites/entrez/?db=gene) and primer 3 designer (http://frodo.wi.mit.edu/primer3-0.4.0/input.htm). qPCR was performed on an Applied Biosystems 7300 PCR system (Bio-Rad Laboratories, Hercules, CA). The genes of interest were normalized to the housekeeping gene following the standard method: C_T_ values of the housekeeping gene were subtracted from the C_T_ values of the gene of interest (ΔC_T_) and normalized to baseline (ΔΔC_T_). All samples were run in duplicate and C_T_ was automatically calculated.

### Tissue preparation

Total protein was isolated from skeletal muscle tissue samples using an electric homogenizer in 20 µL of 0.05 M KPO_4_ buffer (5 mM EDTA, 0.5 mM DTT, 1.15% KCl (w/v)) per milligram of tissue. A protease inhibitor cocktail (Sigma, St. Louis, Missouri) was dissolved in buffer immediately prior to homogenization at a ratio of 1 tablet per 10 mL buffer. Samples were centrifuged at 600 *g* for 10 min at 4°C and the supernatant aliquoted and stored at −80°C. Total protein concentrations were determined using a bicinchoninic acid (BCA) assay (Pierce Biotechnology, Rockford, IL) following manufacturer’s instructions and absorbance measured with a plate spectrophotometer (Bio-Rad Laboratories, Hercules, CA).

### Western blotting

Proteins were separated on SDS-polyacrylamide gels, transferred to Hybond ECL nitrocellulose membrane (Amersham Biosciences, Piscataway, NJ) and blocked with either 5% BSA (w/v) or 2% milk protein in Tris-buffered saline with 0.1% Tween (vol/vol) (TBST). The following commercially available primary antibodies were used: MitoProfile Total OXPHOS Human Cocktail (abcam, Cambridge, MA; ab110411, 1∶1000); Atrogin-1 (abcam; ab67866, 1∶1000); MURF1 (abcam; ab96857, 1∶1000); total ubiquitin (Santa Cruz Biotechnology, Dallas, TX; sc-8017, 1∶1000); NEDD4 (abcam; ab14592, 1∶1000). After washing in TBST, membranes were incubated in either HRP-linked anti-rabbit or anti-mouse IgG secondary antibody (Amersham Biosciences), washed with TBST and developed using ECL (Amersham Biosciences; model no. RPN2106). Membranes were exposed to x-ray film (Biomax XAR; Kodak, Rochester, New York). Films were then scanned with a Dell 920 scanner (North York, ON, Canada) at 300 DPI and saved in TIFF file format. Using Image J v1.40g software (National Institutes of Health, Bethesda, Maryland), background noise was removed and bands in the region of interest were selected for analysis. Individual profile plots were generated and area under the curve measured in arbitrary units (AU). All proteins were normalized for gel loading using Ponceau S.

### Enzyme activity

Maximal enzyme activities were measured with a Cary Bio-300 UV-Vis spectrophotometer (Varian, Inc., Palo Alto, CA). Enzyme activities are expressed as IU · mg of protein^−1^.

#### Citrate synthase (CS)

Activity was measured by mixing 830 µL Tris buffer (0.1M, pH 8.0, 37°C), 100 µL of DTNB (0.5 mg/mL Tris buffer), 10 µL of Acetyl CoA (6.25 mg/mL Tris Buffer) and 10 µL muscle homogenate in 1.5 mL reference and measurement cuvettes. Cuvettes were placed in spectrophotometer (37°C) and the reaction was started by adding 50 µL of oxaloacetic acid (1.22 mg/mL Tris buffer) to the measurement cuvette only, mixing thoroughly. Absorbance was continuously recorded for 2 minutes at 412 nm and the enzyme activity calculated. Activity was normalized to protein content.

#### Cytochrome c oxidase (COX)

Activity was measured by mixing 970 µL KPO_4_ buffer (0.05 M, pH 7.4, 37°C) and 10 µL reduced cytochrome *c* (24 mg/mL KPO_4_ buffer) in 1.5 mL reference and measurement cuvettes. Cuvettes were placed in spectrophotometer (37°C) and the reaction was started by adding 20 µL of muscle homogenate to the measurement cuvette only, mixing thoroughly. Absorbance was continuously recorded for 2 minutes at 550 nm and the enzyme activity calculated. Activity was normalized to protein content.

### Statistical Analysis

Student’s paired t-tests were used to test for differences at baseline for age, height, weight, BMI, body fat percentage, lean body mass, bone mineral content, isometric strength and VO_2peak_ between END>RES and RES>END groups. Two-way repeated measures ANOVAs (group×time) were used to assess if protein levels, enzyme activities, experimental leg mass, isometric strength, aerobic capacity and the linear 2^−ΔΔCT^ data sets for gene expression were statistically different. All analyses were completed using STATISTICA for Windows 5.0 (Statsoft, Tulsa, OK); the threshold for significance was set at P≤0.05. If statistical significance was achieved, Tukey’s honestly significant difference (HSD) post-hoc test was used to determine the significance among the means. All data are presented as mean ± SD.

## Results

### Baseline measurements

There were no differences in average participant age, height, weight, BMI, body fat percentage, lean body mass, bone mineral content, maximum isometric strength (experimental and control leg) or VO_2peak_ between the END>RES and RES>END groups at baseline (BL) (Table 2).

**Table 2:** Subject characteristics before interventions.

	Res-End	End-Res	P value
Age (y)	19±1.3	20±1.6	0.40
Height (cm)	174.2±10.3	168.0±8.6	0.17
Weight (kg)	67.4±16.5	71.4±17.4	0.62
BMI (kg/m^2^)	21.9±2.8	25.4±6.2	0.14
Body fat (%)	24.4±11.2	30.8±12.6	0.74
Lean body mass (kg)	45.9±10.8	44.5±10.1	0.77
Bone mineral content (g)	24.4±11.2	24.4±11.2	0.62
Experimental Leg Maximum Isometric Strength (Nm)	212.6±78.9	238.4±76.7	0.49
Control Leg Maximum Isometric Strength (Nm)	229.4±80.9	248.1±76.5	0.62
VO_2peak_ (ml/kg/min)	39.2±7.4	38.7±6.1	0.88
Males (N)	5	5	
Females (N)	4	4	

Mean ± SD.

### Gene expression changes following acute concurrent exercise

**Figure 3:**
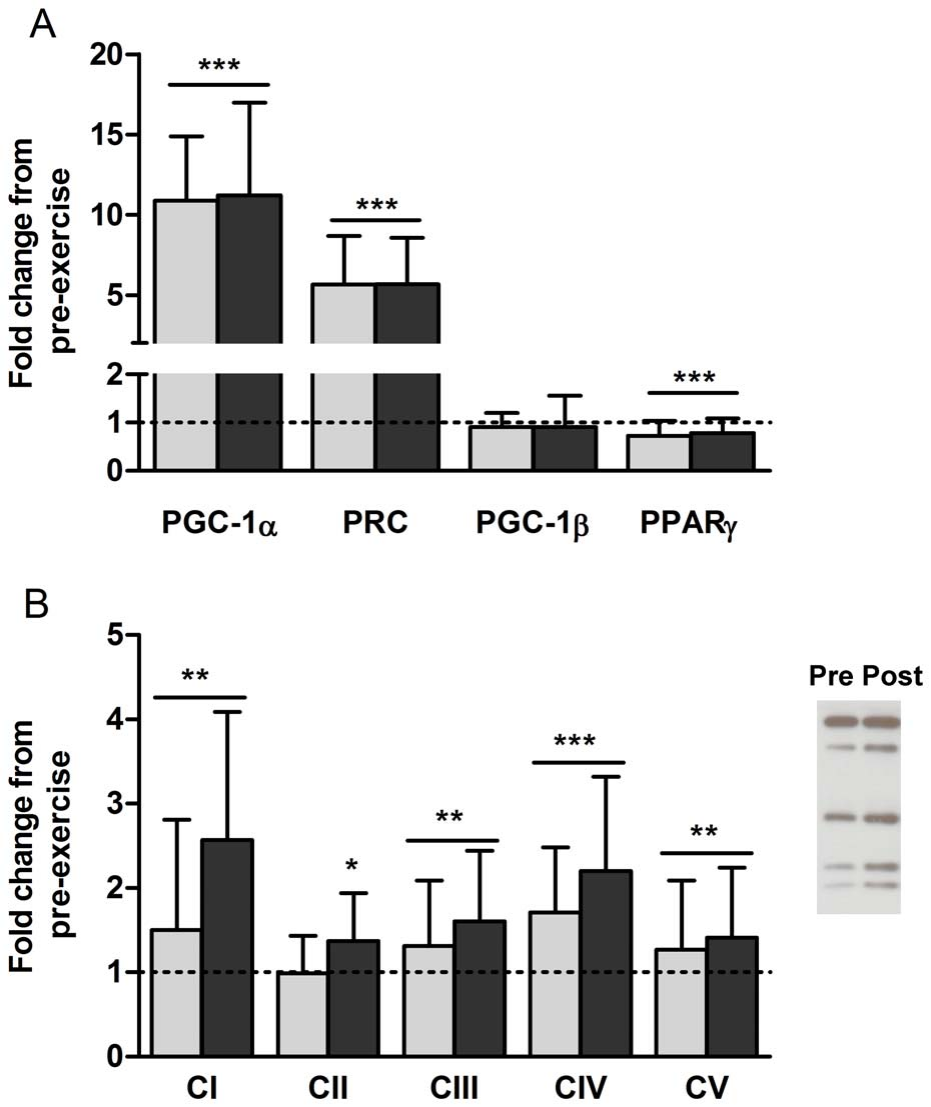
Concurrent exercise alters mRNA content of genes involved in mitochondrial biogenesis and metabolism and increases protein content of subunits of the ETC. A - Gene expression changes in skeletal muscle 3 hours after concurrent rehabilitative exercise for genes involved in aerobic exercise adaption. Peroxisome proliferative activated receptor (PPAR) gamma coactivator-1α (PGC-1α), PGC-1-related coactivator (PRC), PGC-1β, and PPARγ. B - Fold changes in protein content from pre-exercise for subunits of the 5 complexes of the ETC (CI-CV) following 6 weeks of concurrent rehabilitative exercise. Light bars indicate END>RES group, dark bars indicate RES>END group. Representative western blotting image for pre-exercise and post included. *Significant difference from baseline (P<0.05). **Significant difference from baseline (P<0.01). ***Significant difference from baseline (P<0.001). Bar indicates main effect for time and not exercise group. Mean ± SD.

The following genes were measured for their role in aerobic adaptation as regulators of mitochondrial biogenesis: peroxisome proliferative activated receptor (PPAR) gamma coactivator-1α (PGC-1 α), PGC-1β, PPARγ and PGC-1-related coactivator (PRC) (Figure 3A). PGC-1α had the greatest response to acute exercise, increasing its expression level more than 10-fold at 3H. Although not as strongly induced, PRC was increased by almost 570%. Acute PGC-1β expression was not different from pre-exercise level. Only one gene, PPARγ, was expressed at a lower level after acute exercise. The order of exercise did not differentially affect gene expression levels as P values for interactions between group and time ranged between 0.2 and 0.87.

**Figure 4:**
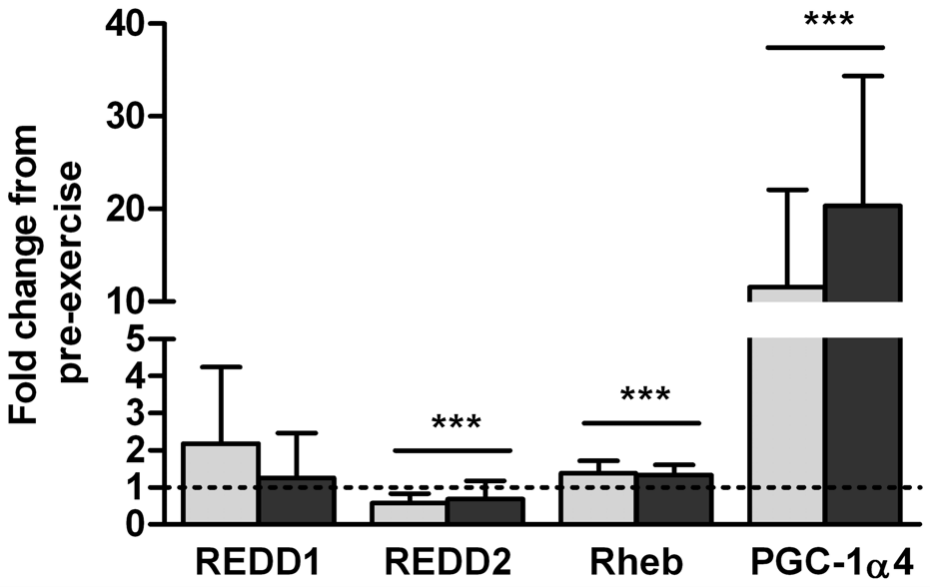
Concurrent exercise alters mRNA content of genes involved in the regulation of mTOR signaling, and proposed IGF-1 and myostatin gene expression. Gene expression changes in skeletal muscle 3 hours after concurrent rehabilitative exercise for genes involved in resistance exercise adaption. Light bars indicate END>RES group, dark bars indicate RES>END group. Regulated in DNA damage 1 (REDD1) and 2 (REDD2), Ras homolog enriched in brain (Rheb), and peroxisome proliferative activated receptor (PPAR) gamma coactivator-1α, isoform 4 (PGC-1α4), ***Significant difference from baseline (P<0.001). Bar indicates main effect for time and not exercise group. Mean ± SD.

To determine whether concurrent exercise affected mRNA involved in hypertrophy, the following genes were measured as they have been described as regulators of Akt/mTOR pathway signaling (Ras homolog enriched in brain (Rheb), regulated in DNA damage 1 (REDD1) and 2 (REDD2)) and expression of IGF1 and myostatin (PGC-1α, isoform 4 (PGC-1α4)). Acute concurrent exercise affected each Akt/mTOR regulator gene differently: REDD1 content was unchanged, REDD2 content was lower, and RheB content was higher. PGC-1α4 had the greatest response to acute exercise, increasing its expression level more than 10-fold at 3H (Figure 4). Order of exercise did not affect gene expression levels as P values for the interaction between group and time for each was greater than 0.77.

**Figure 5:**
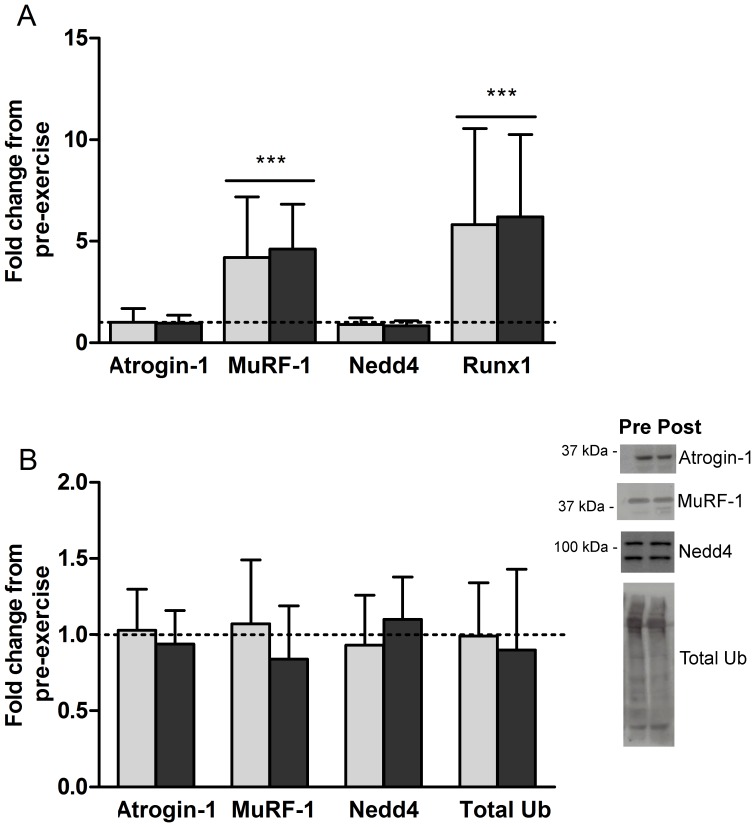
Concurrent exercise acutely increases mRNA content of genes involved in the regulation of muscle atrophy but does not affect chronic protein content of selected E3 ligases or total ubiquitination. A - Gene expression changes in skeletal muscle 3 hours after concurrent rehabilitative exercise for genes involved in regulation of protein breakdown. Atrogin-1, muscle-specific RING finger-1 (MuRF-1), neural precursor cell expressed developmentally downregulated protein 4 (Nedd4), and Runt-related transcription factor 1 (Runx1). B - Fold changes in protein content from pre-exercise levels for muscle specific (Atrogin-1 and MuRF-1) and ubiquitously expressed (Nedd4) E3 ligases, and total ubiquitination (Total Ub) following 6 weeks of concurrent rehabilitative exercise. Light bars indicate END>RES group, dark bars indicate RES>END group. Representative western blotting images for pre-exercise and post included. ***Significant difference from baseline (P<0.001). Bar indicates main effect for time and not exercise group. Mean ± SD.

The genes selected as regulators of protein degradation either induce (Atrogin-1, muscle-specific RING finger-1 (MuRF-1), neural precursor cell expressed developmentally downregulated protein 4 (Nedd4)) or attenuate (Runt-related transcription factor 1 (Runx1)) skeletal muscle atrophy. There was no difference in the levels of Atrogin-1 and Nedd4 mRNA 3 hours after exercise (Figure 5A). MuRF-1 and Runx1 were both strongly induced by exercise, increasing to more than 500 and 700% of baseline levels respectively. Again, order of exercise did not affect gene expression levels as the P value for the interaction between group and time was greater than 0.6 for all.

### Protein expression changes following 6-weeks of rehabilitative concurrent exercise

Protein content of subunits of complexes I to V (CI –CV) of the electron transport chain (ETC) were measured upon completion of the 6 week rehabilitative exercise program and expressed relative to pre-exercise values. Although there was a main effect for increased protein content with time for all five complexes, CII was the only complex significantly different between the exercise protocols with RES>END higher than END>RES (P = 0.037) (Figure 3B).

Protein content of the muscle specific E3 ligases Atrogin-1 and MuRF-1, the ubiquitously expressed E3 ligase Nedd4 and total ubiquitination were measured after rehabilitative exercise. None were different from baseline nor were any differentially affected by order of concurrent exercise (Figure 5B).

### Effect of concurrent exercise on mitochondrial enzyme activity

**Figure 6:**
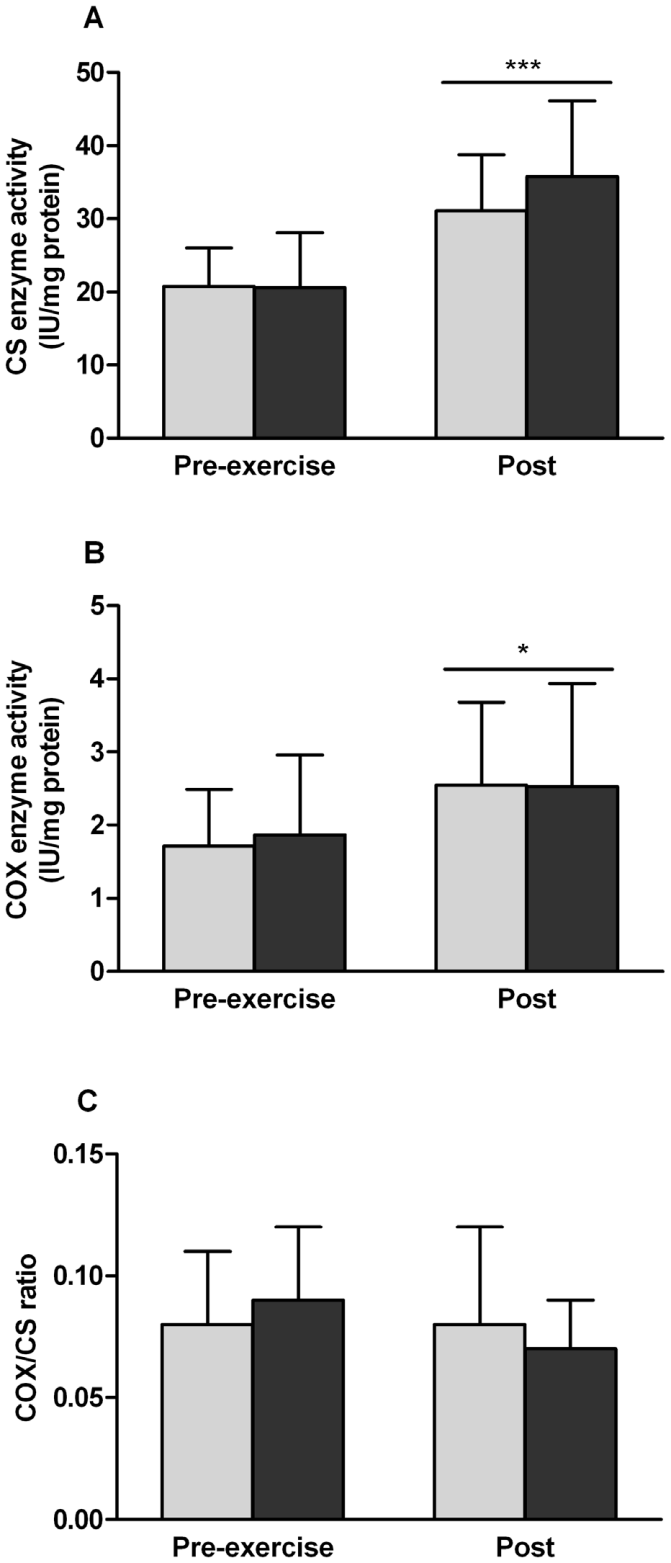
Concurrent exercise increases mitochondrial enzyme activity. Enzyme activities in skeletal muscle before (Pre-exercise) and after 6 weeks of concurrent rehabilitative exercise (Post). A - citrate synthase (CS), B - cytochrome *c* oxidase (COX) and C – COX/CS ratio. Light bars indicate END>RES group, dark bars indicate RES>END group. *Significant difference from baseline (P<0.05). ***Significant difference from baseline (P<0.001). Bar indicates main effect for time and not exercise group. Mean ± SD.

CS and COX maximal activities were 61% (P<0.001) and 51% (P = 0.002) higher after 6 weeks of concurrent exercise, respectively (Figure 6A and 6B). The COX/CS ratio was unaffected by the exercise protocol (Figure 6C). Order of concurrent exercise did not affect any of the measurements or calculations.

### Performance changes after rehabilitative concurrent exercise

**Figure 7:**
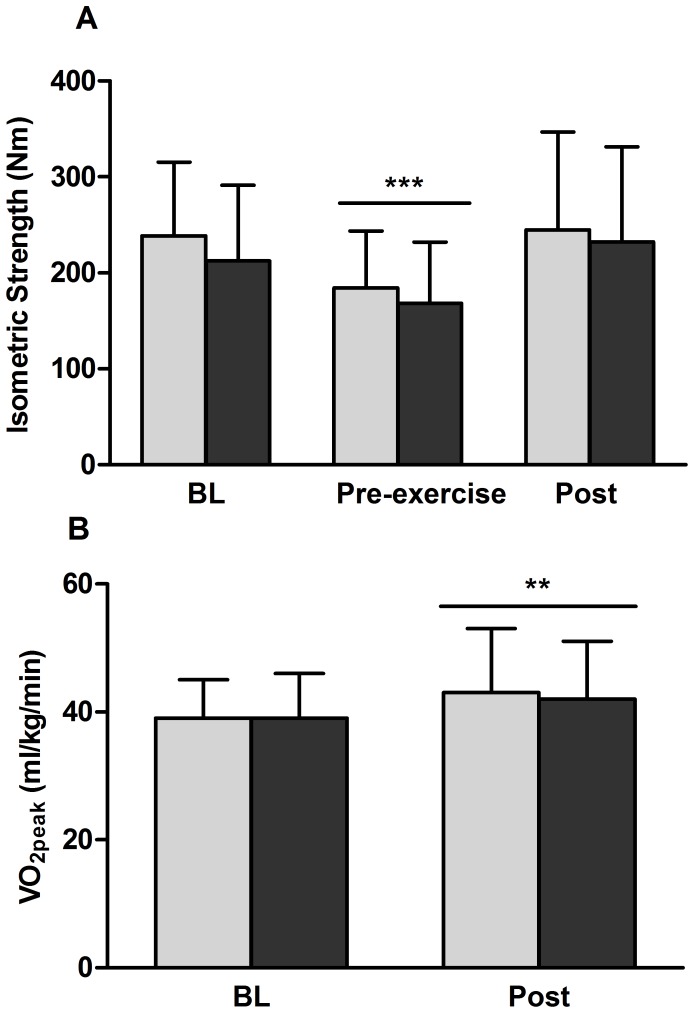
Concurrent exercise recovers maximum isometric strength loss induced by disuse and increases aerobic performance. A - Values for maximum isometric strength of the knee extensors before immobilization (BL), before exercise (pre-exercise) and after 6 weeks of rehabilitative concurrent exercise (Post). B - peak oxygen consumption (VO_2peak_) before immobilization (BL) and after 6 weeks of concurrent exercise (Post). Light bars indicate END>RES group, dark bars indicate RES>END group. **Significant difference from baseline (P<0.01). ***Significant difference from baseline (P<0.001). Bar indicates main effect for time and not exercise group. Mean ± SD.

Following 6 weeks of rehabilitative exercise, maximum isometric strength of the knee extensors of the immobilized leg increased by 35% from pre-exercise values (P<0.001) regaining the strength lost by immobilization (Figure 7A). Maximum isometric strength of the control leg was unchanged over the course of the study (results not shown). Peak aerobic capacity significantly increased by 9% between the baseline measures taken before immobilization and after rehabilitative exercise (P = 0.006) (Figure 7B). The order of concurrent exercise did not affect the increases measured in either maximum isometric strength or VO_2peak_.

### Muscle mass changes after rehabilitative concurrent exercise

Two weeks of immobilization significantly reduced the mass of the experimental leg from 8.6 kg to 8.4 kg (P = 0.04). Six weeks of concurrent exercise increased the leg mass, surpassing the amount of tissue lost by disuse by 0.3 kg to reach 8.9 kg (P<0.001).

## Discussion

Resistance and aerobic exercises are rehabilitation therapies used to regain losses in strength and aerobic capacity that typically occur during injury, illness or chronic disuse of skeletal muscle. Performing these exercises together in the same session as concurrent exercise typically increases both strength and aerobic function; however, some research suggests that interference in the signaling patterns for adaptation favor one phenotypic outcome, depending on the order in which the exercise modes are performed [27], [30]. As such, we examined the effects of alternating the order of exercise in concurrent training protocols as a rehabilitation strategy for preferentially improving strength or aerobic capacity following 2 weeks of induced hypodynamia. Contrary to our hypothesis we did not find differences in the gene expression patterns, most protein content changes, and the increases in isometric strength or peak aerobic capacity between groups that completed concurrent exercise protocols in which the first mode was either endurance exercise or resistance exercise.

Concurrent exercise is an effective and efficient method of reversing losses in strength and aerobic capacity that may result from prolonged skeletal muscle disuse. Regardless of the order in which the exercise modes are performed, we observed almost identical improvements in both maximum isometric strength and VO_2peak_ after 6 weeks of rehabilitative concurrent exercise. A meta-analysis of 21 studies examining concurrent training and the interferences between aerobic and resistance exercise concluded that concurrent training provides similar respective increases in strength and VO_2max_ as those found with resistance and aerobic exercise alone [25]. They reported that the mean overall effect size (ES) for increasing strength using resistance training alone (ES = 1.76, 95% CI: 1.34, 2.18) was not significantly different from concurrent exercise (ES = 1.44, 95% CI: 1.03, 1.84) [25]. Similarly, the improvements in VO_2max_ following either endurance only or as part of a concurrent training model were almost identical; 1.37 (95% CI: 0.85, 1.88) and 1.41 (95% CI: 0.93, 2.95) respectively [25]. Although it we do not know whether greater gains in either metric could have been achieved by focusing on one exercise mode only for rehabilitation, we did not observe preferential adaptation based on the order of the exercises performed.

As highlighted by the above meta-analysis, many studies have compared the outcome metrics and signaling patterns following concurrent and single mode exercise protocol. However, none have tested the benefits of concurrent exercise as a mechanism for muscle rehabilitation following disuse atrophy. Additionally, studies examining if outcome measures are affected by the order in which the modes of exercise are performed during a concurrent exercise session are limited. Two studies that were found used trained males in 2 week crossover study designs to compare acute gene expression and protein signaling after single mode and concurrent exercise [27], [30]; the primary difference between them being that one used maximum sprint intervals (10×6 s) [30] and the other submaximal cycling (30 min @ 70% VO_2max_) [27]. They reported a greater increase in MuRF-1 mRNA content when resistance exercise was performed before both endurance or sprint exercise [27], [30], a greater increase in insulin-like growth factor-1 (IGF-1) and hexokinase II (HKII) mRNA when resistance exercise preceded endurance exercise [27] and attenuated expression of a regulator of mitochondrial biogenesis (PGC-1α) when resistance exercise preceded sprint but not endurance exercise [27], [30]. These results led the authors to conclude that the acute response to concurrent exercise is affected by the order in which the exercises are performed [27], [30]. We, however, did not find differential expression patterns in any of the genes we selected, including MuRF-1 or PGC-1α, although both were induced by concurrent exercise. Reasons for the different results may be due to differences in exercise protocols, timing of exercise and biopsies, or training status (detrained vs. well-trained) of the individuals at the time of testing. However, our conclusion that order of exercise did not affect mRNA content of any of the genes we measured agrees with the similar improvements in outcome performance metrics between RES>END and END>RES groups.

We also sought to determine whether altering the order of exercise had an effect on other genes involved in mitochondrial biogenesis. Originally identified for its role in oxidative metabolism in brown adipose tissue, PGC-1α translocates to the nucleus and mitochondria of skeletal muscle following exercise to induce the transcription of nuclear and mitochondrial DNA-encoded mitochondrial genes [31], [32]. Two additional coactivators of mitochondrial biogenesis are PRC [33] and PGC-1β [34]. Acting in a similar manner to PGC-1α, PRC expression is increased with endurance exercise [35], [36] and enhanced when performed concurrently with resistance exercise [37]. To the contrary, chronic endurance exercise appears to attenuate resting PGC-1β protein content [38] though acute exercise does not affect mRNA values [39]. PPARγ, although not abundantly expressed in skeletal muscle, is a regulator of insulin signaling and metabolism as it promotes molecular storage of energy [40], [41]. Elevated muscle PPARγ mRNA expression has been measured with obesity and type II diabetes and is thought to negatively affect insulin resistance [42]. Taken together, the expression changes of PGC-1α, PRC, and PPARγ all support a modification to the transcriptome to increase mitochondrial content and function after concurrent exercise. In support of this, we measured higher protein content of all five complexes of the electron transport chain, higher CS and COX enzyme activity and higher VO_2peak_ at the end of training. Although statistical analyses reported similar increases in VO_2peak_, CS enzyme activity, COX enzyme activity, and 4 of the 5 ETC complexes between RES>END and END>RES groups, there may be trend for greater protein content of the ETC complexes and CS enzyme activity when resistance exercise followed endurance. Further work is needed to determine if this would become significant at different time points or using different exercise intensities and modes.

A major regulator of the protein synthesis associated with hypertrophy is the protein kinase, mammalian target of rapamycin (mTOR) [43]–[45]. Once activated, mTOR phosphorylates substrates involved in the translation of mRNA for protein accretion [45], [46]. Activation and regulation of mTOR occurs in response to many stimuli that include but are not limited to growth factors, nutrients, cellular stress and a number of small proteins [45]. Rheb is a small GTPase that positively regulates mTOR [43], [45], [47] while REDD1 and REDD2 negatively regulate mTOR [45], [47]–[49]. The lack of change in REDD1 mRNA expression, reduced REDD2 expression and increased Rheb expression suggest a coordinate response for positive regulation of mTOR signaling by concurrent exercise, that was unaffected by order in which the modes of exercise were performed. The specific actions of PGC-1α4, a recently identified isoform of PGC-1α, remain to be well-defined. This splice variant was first described as important in the adaptation to strengthening exercise rather than aerobic exercise as its target genes were important for hypertrophy (IGF-1 and myostatin), and was stimulated by resistance not endurance exercise [32]. However, subsequent work has not supported this unique response as both PGC1α and PGC1α4 can be induced by either resistance or endurance exercise separately [50], [51]. Our data suggests that PGC-1α4 can be highly upregulated immediately following concurrent exercise and unaffected by order of exercise mode. Together, these early gene responses are supported by the increased strength and lean tissue mass following rehabilitation that was not different between RES>END and END>RES groups.

The ubiquitin proteasome pathway is the primary cellular mechanism for protein breakdown [52]. Targeting of specific proteins for degradation by the proteasome is achieved through binding of the chains of the regulatory protein ubiquitin (polyubiquitination) by E3 ligases [53]. Muscle contains two specific E3 ligases, Atrogin-1 and MuRF-1, whose gene expression levels are higher following unweighting and immobilization in humans [53]–[55]. Additionally, as part of the breakdown initiated as part of the remodeling process [56], [57], expression of both of these genes may be increased following unaccustomed resistance exercise [58], [59]. Unlike the muscle specific expression of Atrogin-1 and MuRF-1, Nedd4 is a ubiquitously expressed E3 ligase that is also upregulated in muscle following unloading and denervation in rodents [60], [61] and human patients with severe COPD [62]. Negative regulation of skeletal muscle atrophy is also necessary to conserve muscle mass in times of disuse [63], [64]. One of the first autophagy suppressors described was Runx1 [63]. Although the exact mechanism for its suppression is unclear, it may be through the modulation of FoxO3 [65], a mediator of Atrogin-1 and MuRF-1 mRNA expression [66]. Our results indicate that MuRF-1 and Runx1 were highly induced by concurrent exercise following immobilization. As positive and negative regulators of autophagy, the ultimate outcome of their signaling is unknown, although the increase in muscle mass suggests that they may have regulated muscle remodeling. The lack of change in Atrogin-1 and Nedd4 content at 3H may have been a result of the time selected for measurement, or induction following immobilization may not have been possible. Protein levels of Atrogin-1, MuRF-1, Nedd4 and total ubiquitination were not different when measured after 6 weeks of rehabilitative exercise, likely because the muscle had habituated to the exercise regime and muscle protein synthesis superseded breakdown to aid in hypertrophy. None of our measurements of markers of autophagy were affected by the order of exercises performed, suggesting that alternating concurrent exercises may affect autophagy similarly when used as a modality for rehabilitation.

## Conclusion

Concurrent exercise is an effective and efficient rehabilitation protocol for regaining lost strength and muscle mass associated with prolonged disuse. When performed immediately after one another, the order of exercise does not differentially influence the chronic mitochondrial enzyme activities changes or increases in maximum strength or aerobic capacity. Although the genes and most proteins we selected were not different between groups, adaptation to multimode exercise involves coordination of a myriad of unmeasured signaling pathways that may have been different. However, in the absence of an effect on performance any single difference may be acute or opposed by another. As a result, we have shown for the first time that concurrent exercise sessions provide a reproducible intervention for the clinical treatment of short term muscle atrophy. This is important as the translation of the adaptations following exercise interventions in health people to those in need of rehabilitation is questionable.
